# Nanoencapsulated Essential Oils with Enhanced Antifungal Activity for Potential Application on Agri-Food, Material and Environmental Fields

**DOI:** 10.3390/antibiotics10010031

**Published:** 2021-01-01

**Authors:** Magdaléna Kapustová, Giuseppe Granata, Edoardo Napoli, Andrea Puškárová, Mária Bučková, Domenico Pangallo, Corrada Geraci

**Affiliations:** 1 Institute of Molecular Biology, Slovak Academy of Sciences, Dúbravská cesta 21, 84551 Bratislava, Slovakia; magdalena.kapustova@savba.sk (M.K.); andrea.puskarova@savba.sk (A.P.); maria.buckova@savba.sk (M.B.); 2 Istituto Chimica Biomolecolare–Consiglio Nazionale delle Ricerche, Via Paolo Gaifami 18, 95126 Catania, Italy; giuseppe.granata@icb.cnr.it (G.G.); edoardo.napoli@icb.cnr.it (E.N.)

**Keywords:** antifungal activity, essential oils, nanoencapsulation, poly(ε-caprolactone), *Origanum vulgare*, *Thymus capitatus*

## Abstract

Nanotechnology is a new frontier of this century that finds applications in various fields of science with important effects on our life and on the environment. Nanoencapsulation of bioactive compounds is a promising topic of nanotechnology. The excessive use of synthetic compounds with antifungal activity has led to the selection of resistant fungal species. In this context, the use of plant essential oils (EOs) with antifungal activity encapsulated in ecofriendly nanosystems could be a new and winning strategy to overcome the problem. We prepared nanoencapsules containing the essential oils of *Origanum vulgare* (OV) and *Thymus capitatus* (TC) by the nanoprecipitation method. The colloidal suspensions were characterized for size, polydispersity index (PDI), zeta potential, efficiency of encapsulation (EE) and loading capacity (LC). Finally, the essential oil nanosuspensions were assayed against a panel of fourteen fungal strains belonging to the Ascomycota and Basidiomycota phyla. Our results show that the nanosystems containing thyme and oregano essential oils were active against various fungal strains from natural environments and materials. In particular, the minimum inhibitory concentration (MIC) and minimum fungicidal concentration (MFC) values were two to four times lower than the pure essential oils. The aqueous, ecofriendly essential oil nanosuspensions with broad-spectrum antifungal activity could be a valid alternative to synthetic products, finding interesting applications in the agri-food and environmental fields.

## 1. Introduction

Fungi are ubiquitous microorganisms that can colonize several natural environments, equipped with enzymatic machinery enabling the degradation of various types of materials and matrices [1]. Members of the genus *Aspergillus*, such as *A. fumigatus* and *A. flavus*, together with *Penicillium rubens*, can be isolated from indoor environments and can colonize and damage cellulolytic substrates such as archival documents and books [2,3]. The fungi of the genus *Cladosporium* are well known as phytopathogenic microorganisms able to infect different kinds of plants [4]. *Penicillium citrinum* is recognized mainly as a citrus fruit pathogen, but occasionally it also occurs in tropical spices and cereals [5]. Other fungi, such as members of the genera *Geotrichum*, *Mucor* and *Fusarium*, can be isolated from various foods where they can release dangerous mycotoxins [6,7].

There are fungi typically from soil, such as *Purpureocillium* and *Exophiala*, and certain types of them can successfully inhabit stones and be responsible for bioweathering phenomena [1,8]. Different species, mainly belonging to the phylum Basidiomycota, are considered to be wood-decay fungi such as *Pleurotus eryngii*, *Bjerkandera adusta* and *Phanerochaete chrysosporium* [9]. The wood-decay fungi can also lead to the biodeterioration of wood components commonly used in construction and may impose serious problems on building stability [10]. Finally, many of the fungi mentioned above can cause various fastidious health complications and, in some cases, also serious pathogenicity [11]. Therefore, it is necessary to implement a series of precautions for their control and inhibition on various kinds of surfaces (especially in an indoor environment) and in food processing.

Over the decades, the massive use of synthetic antifungal products induced resistance phenomena in a large number of fungal species. In this direction, the use of substances derived from plants, such as essential oils, can be considered a valid alternative that better meets the wishes of consumers increasingly oriented toward natural remedies [12]. Essential oils (EOs) are phytocomplexes obtained from aromatic plants by hydrodistillation or steam distillation. Their chemical composition is very complex due the presence of a large variety of volatile compounds, mainly terpenes. Monoterpenes and sesquiterpenes are present in the form of hydrocarbons, alcohols, aldehydes, ketones, esters, ethers, peroxides and phenols. Phenylpropane derivatives occur less frequently than the aforesaid terpenes [13]. The EO chemical composition is influenced by the geographical position, environment condition, stage of ripening and extraction technique. More than half of the essential oils from Lamiaceae family plants have good antifungal activity (minimum inhibitory concentrations (MICs) < 1000 µg/mL) [14]. As reported by Rao et al. [15], terpenes and terpenoids are known to exhibit intense antifungal activity. Their action mechanism is multitarget and does not favor the appearance of resistance fungal strains [16,17].

Unfortunately, EOs are lipophilic compounds, easily degradable by the effects of oxygen, light, moisture and temperature. Nanoencapsulation is a valid strategy to overcome these obstacles. This technology allows for the protection the essential oils from thermal and photodegradation phenomena, increasing their solubility in aqueous environments, masking their flavor and improving their bioaccessibility and bioavailability [18]. The subcellular size and relative larger surface area per unit volume enhances EO concentration in the zone where the microorganisms are preferentially located, such as water-rich phases or liquid–solid interfaces [19].

The nanocapsules, prepared by interfacial deposition of the preformed polymer (nanoprecipitation), represent an effective method to obtain robust nanosystems suitable (by a feasible scale-up process) for applications in various fields, ranging from medicine, health and agri-food to the environment [20]. In these systems, the EO is located in the inner core, surrounded by a polymeric wall.

In this study, nanocapsules based on the biodegradable and biocompatible poly(ε-caprolactone) (PCL) polymer were prepared. The nanocapsules were loaded with commercial EOs of *Origanum vulgare* (OV) and *Thymus capitatus* (TC), both having known antifungal activity [21,22,23]. The antifungal ability of the encapsulated and free EOs were assayed against a panel of fourteen different fungi belonging to the Ascomycota and Basidiomycota phyla. These fungi are usually responsible for the biodeterioration and biodegradation of different materials and for the contamination of food, causing damage to human health.

The aim of this work is the preparation of aqueous, ecofriendly nanosuspensions with effective and broad-spectrum antifungal activity for potential applications on different natural matrices and materials.

## 2. Results

### 2.1. Chemical Composition of Essential Oils

Thyme and oregano essential oils are, generally, characterized by a large amount of monoterpenes (both hydrocarbons and oxygenated), reaching almost 90% of the whole oil. The two main phenolic monoterpenes, thymol and carvacrol, occur more frequently, often accompanied by their biogenetically correlated compounds *p*-cymene and γ-terpinene [13,24]. Thymus is probably one of the most taxonomically complex genera of its family. Several studies confirmed that the two most common chemotypes are thymol and carvacrol [25], followed by less common non-phenolic chemotypes [26]. As reported in materials and methods section, the two EO samples of this study were commercial. The chemical composition of our oregano sample has already been published in a previous study with different purposes from this one [27], while the chemical composition of our commercial thyme EO is published here for the first time. The two analytical results are listed together in Table 1 for easier comparison by readers. The samples subjected to this study had chemical profiles quite typical of the *Origanum vulgare* thymol and carvacrol chemotype and the *Thymus capitatus* carvacrol chemotype of essential oils. Gas chromatography (GC) techniques, coupled with a flame ionization detector (FID) and a mass spectrometer (MS), allowed the identification of more than 35 components, covering up to 99% and 98% of the total oil compositions, respectively. Table 1 shows the details of the chemical compositions, listing only the components with a % > 0.05. The most represented class for both samples was that of oxygenated monoterpenes (68.7% and 72.9%, respectively), followed by hydrocarbon monoterpenes (30.0% and 22.3%, respectively). The sum of these classes in the oregano sample reached 96.7%, while in the thyme sample it reached 95.1%. For both samples, sesquiterpenes and other components were below 4%. The common feature of these two oils was to have three main components which alone covered more than 80% of the whole composition. The oregano essential oil profile was characterized by the presence of carvacrol (36%) and thymol (25%) as the main compounds, followed by *p*-cymene (22%). All other compounds were below 5%. The thyme essential oil profile was dominated by a high amount of carvacrol (almost 70%), followed by *p*-cymene (9%) and γ-terpinene (8%). The thymol percentage was negligible (0.5%), as were the percentages of all other compounds, with the exception of β-caryophyllene, which reached 2.6%.

### 2.2. Physicochemical Characterization of Essential Oil-Loaded Nanocapsules (EO-NCs)

In a previous work, we reported the encapsulation of thyme and oregano essential oils in PCL nanocapsules [28]. Unlike the previous work, here we used commercial essential oils having different compositions of volatile components. This offered numerus advantages, including the lower cost of preparation and the standardization in the compositions of the EOs. In this manner, the obtained nanocapsules could potentially be more attractive for industrial sectors. Table 2 shows the reported values of the physicochemical parameters (z-average diameter, polydispersity index (PDI), zeta potential (*ζ*), encapsulation efficiency (EE) and loading capacity (LC)) which allowed us to characterize the nanocapsules containing thyme and oregano essential oils (TC-NCs and OV-NCs, respectively). 

**Figure 1:**
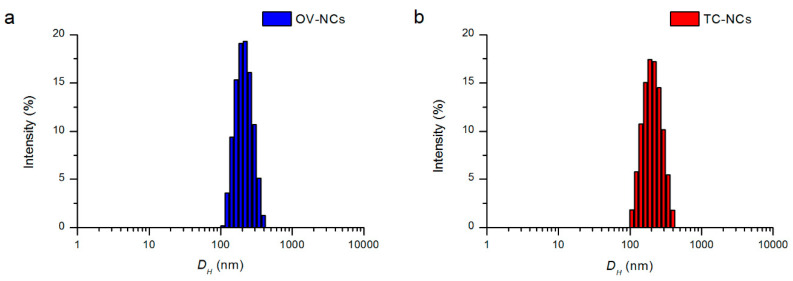
I-weighted distribution of the hydrodynamic diameter (*D_H_*) of (**a**) OV-NCs and (**b**) TC-NCs.

The z-average diameters of 198 ± 3 nm and 200 ± 3 nm for the TC-NCs and OV-NCs, respectively, were in agreement with the nanometric structures of the nanocapsules. Low PDI values for both nanocapsules pointed out a narrow size distribution of nanoparticles and the presence in aqueous solution of monodisperse nanosystems (Figure 1). Negative zeta potentials of −11 and −10 mV for the TC-NCs and OV-NCs, respectively, were very similar to those obtained for other stable PCL nanocapsules [28,29]. The percentages of EE and LC were high, with values of 84 ± 6 and 52 ± 3 for the TC-NCs and 80 ± 9 and 51 ± 4 for the OV-NCs. The prepared TC-NC and OV-NC aqueous nanosuspensions showed a total essential oil content of 5.7 ± 0.3 and 5.8 ± 0.4 mg/mL, respectively.

### 2.3. Antifungal Activities of Pure EOs and EO-NCs

**Figure 2:**
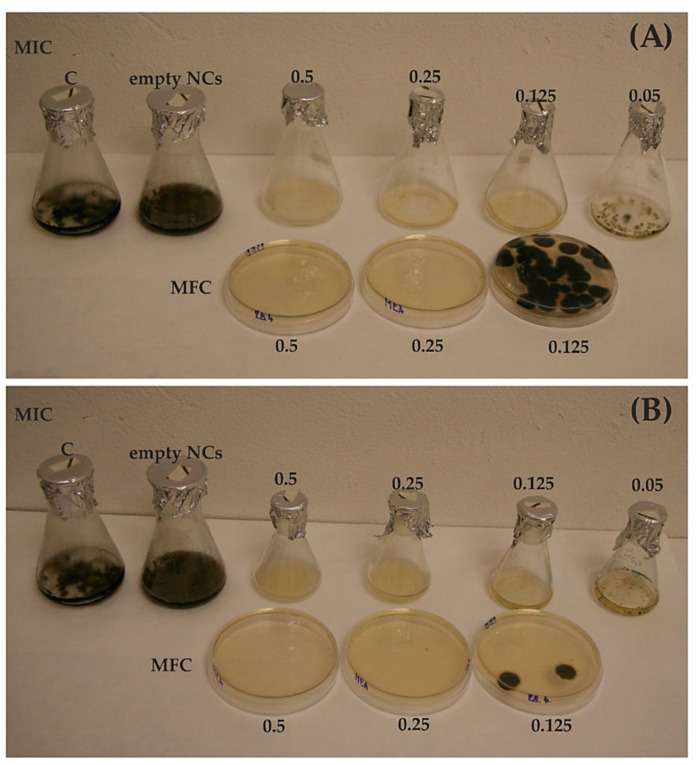
Example of MIC and MFC assays with the isolate *Cladosporium herbarum*. (**A**) Results using a nanoencapsulated oregano EO. (**B**) Results using a nanoencapsulated thyme EO. C = control. The range of nanoencapsulated EO concentrations used was 0.05–0.5 mg/mL.

The screening of encapsulated essential oils (EO-NCs) against a panel of fungal strains (Table 3) showed marked antifungal activity (Table 4 and Figure 2). The obtained results evidenced that EO-NCs inhibited the growth of assayed fungi at s MIC values ranging from 0.125 to 0.25 mg/mL. These concentrations were two to four times lower than those observed for pure EOs. For both EO-NCs, an MIC of 0.125 mg/mL was effective for inhibiting the growth of *A. fumigatus*, *C. aggregatocicatricatum*, *C. herbarum* and *P. eryngii*. In addition, this concentration for OV-NCs was also enough to inhibit the fungus *Bjerkandera adusta*.

We also evaluated the fungicidal effect of EO-NCs versus the panel of fourteen fungal strains. In particular, both the TC-NCs and OV-NCs had minimum fungicidal concentrations (MFCs) of 0.25 mg/mL against *A. fumigatus*, *C. aggregatocicatricatum*, *C. herbarum* and *P. eryngii*, while having an MFC of 0.5 mg/mL against *A. flavus*, *P. rubens*, *P. citrinum*, *F. oxysporum*, *G. candidum*, *M. circinelloides*, *E. xenobiotica*, *P. lilacinum* and *P. chrysosporium*. The MFC values of the EOs were two to four times higher than the MFCs of the EO-NCs on the tested strains. The empty NCs revealed no activity on the fungal growth.

## 3. Discussion

The current study evaluated the antifungal activity of TC-NCs and OV-NCs against selected fungal strains that contaminate different natural matrices, indoor air and materials (Table 3). The results evidenced that both TC-NCs and OV-NCs showed in vitro antifungal activity against all tested isolates with an MIC range of 0.125–0.25 mg/mL (Table 4). This activity is two to four times greater than that of pure oils. These results highlight the effectiveness of EO encapsulation in a nanometric structure. In fact, the nanocapsules were able to protect essential oils from degradation phenomena and increase the transport and diffusion mechanisms of essential oils through the cell membrane [30].

Moreover, the same MIC and MFC values were observed for both nanocapsule suspensions in respect to the different strains, with the one exception of the *Bjerkandera adusta* strain, against which the OV-NCs showed more activity than the TC-NCs. The results were imputable to the chemical compositions of commercial essential oils. Although the main bioactive oxygenated monoterpenes (thymol and carvacrol) were present in different quantities in the two essential oils, the sum of their relative percentages was of the same order of magnitude (Table 1). Generally, the growth-inhibiting effects were attributable to the major components, thymol and carvacrol, but the bioactive monoterpene hydrocarbons, *p*-cymene and γ-terpinene present in both essential oils in non-negligible quantities could have given a valuable contribution to the antifungal properties [31].

Regarding the physicochemical characterization of the nanocapsules, the TC-NCs and OV-NCs showed nanodimensions and good PDIs indicating the presence of monodisperse species. This characteristic is crucial for achieving optimal biological activity results [32]. Acidic residues on the PCL polymer and the presence of tween 80 (a neutral surfactant covering the nanoparticle wall) were in agreement with the observed low and negative values of the zeta potential. Moreover, these values were very similar to those previously reported by us [28,29] and by other authors [33,34] for stable poly(ε-caprolactone) nanocapsules.

The good encapsulation in the range of 80–84% and the loading capacity of 51–52% highlighted the ability of the PCL to effectively encapsulate essential oils. Furthermore, the ease of preparation of these nanosystems and their stability in aqueous environments could make them useful in different application fields.

PCL is a biocompatible and biodegradable polymer approved by the Food and Drug Administration for drug delivery application. It is a polymer with a low cost and high hydrophobicity and stability. Moreover, PCL is characterized by slow degradation, which makes it potentially more suitable than other polymers, such as polyglycolide (PGA) and poly D, L-lactide (PDLA), when a slow release of the bioactive is required [35].

We have reported here the first example of PCL-based nanocapsules containing essential oils with more enhanced antifungal activity than pure essential oils against a panel of fourteen fungal strains. In the literature, the antifungal activity of essential oils encapsulated in different nanocarriers was also reported. There are many examples of encapsulated EOs in chitosan nanostructures with antifungal activity [36], the majority of which were obtained by the emulsion ionic gelation technique. In a recent work, Hasheminejad et al. [37] demonstrated that clove essential oil encapsulated by chitosan nanoparticles inhibited the growth of *Aspergillus niger*, isolated from spoiled pomegranate, at a concentration of 1.5 mg/mL. Instead, against *Aspergillus flavus*, Khalili et al. [38] reported an MIC value of 300 mg/L by using a chitosan benzoic acid nanogel containing thyme essential oil under sealed conditions. *Fusarium graminearum* was inhibited by a chitosan-encapsulating *Cymbopogon martini* essential oil [39], with an MIC value of around 0.42 mg/mL.

Nanoemulsions of oregano EO, prepared by Bedoia-Serna et al. [40], showed antifungal effects against *Cladosporium* sp., *Fusarium* sp. and *Penicillium* sp. The authors found that for *Fusarium* sp., unlike with *Penicillium* sp., the encapsulation of the oregano essential oil enhanced its antifungal effect.

β-Cyclodextrin was also used to entrap essential oils with antifungal activity through the formation of an inclusion complex. Capsules of β-cyclodextrin containing clove and Mexican oregano EO were active against *Fusarium oxysporum* [41]. An inclusion complex based on β-cyclodextrin and *Litsea cubeba* EO presented effective antifungal activity against three main fungi in citrus, including *Penicillium digitatum*, *Penicillium italicum* and *Geotrichum citri-aurantii* [42].

The antifungal activity of inorganic nanoparticles loaded with essential oils was reported by Weisany et al. In particular, the authors showed that encapsulation of *Thymus daenensis* and *Anethum graveolens* in silver nanoparticles enhanced their fungicidal activity against the plant pathogen *Colletotrichum nymphaeae* [43]. The same authors proved that copper nanoparticles containing thyme and dill EOs were able to reduce the mycelium growth and strongly inhibit the germination of conidia of *Colletotrichum nymphaeae* [44].

Unexpectedly, in the literature, no example of nanostructured systems containing essential oils effective against the fungi *Mucor circinelloides*, *Exophiala xenobiotica*, *Purpureocillium lilacinum*, *Pleurotus eryngii*, *Bjerkandera adusta* or *Phanerochaete chrysosporium* has been reported.

Although the antibacterial activity of EO nanocapsules based on PCL have been evaluated [28,45,46], less interest has been paid to their antifungal activity. The co-encapsulation of chloramphenicol with lemongrass essential oil in PCL-Pluronic composite nanocapsules was reported by Wang et al. [47]. This nanosystem showed considerably enhanced activity against methicillin-resistant *Staphylococcus aureus* and *Candida* sp. Tea tree essential oil loaded in PCL nanocapsules exhibited antifungal activity against *Trichophyton rubrum*, an etiological agent of superficial human mycosis [48].

In light of this, our results could provide a valuable contribution to the use of encapsulated essential oils as effective natural fungicides, as an alternative to the synthetic ones responsible for increasing resistant fungal strains.

## 4. Materials and Methods

All solvents were of analytic grade. In order to prepare the essential oil-loaded nanocapsules, sorbitan monostearate (SM) and poly(ε-caprolactone) (PCL) (Mn 45000) were obtained from Sigma-Aldrich (Milan, Italy) and polysorbate 80 (Tween 80) was obtained from Fisher Chemical (Fisher Scientific, Geel, Belgium). Water Chromasolv Plus for HPLC (Honeywell Riedel-de-Haën, Seelze, Germany) was used. Commercial essential oils of oregano and thyme were provided by Esperis S.p.A., (Milan, Italy) and by Flora s.r.l. (Lorenzana, Pisa, Italy), respectively. A standard mix of *n*-alkanes C_9_–C_22_ was purchased by Alltech (Italy).

### 4.1. Characterization of Essential Oils by Gas Chromatography–Flame Ionization Detection (GC–FID) and Gas Chromatography–Mass Spectrometry (GC–MS)

GC–FID and GC–MS analysis were performed on a Shimadzu GC-17A and a Shimadzu GCMS-QP5050A, respectively. For both analyses, the same fused silica capillary column (Supelco SPBTM-5 15 m, 0.1 mm, 0.1 mm) was used.

The operating conditions for both runs were as follows: injector temperature 250 °C; detector temperature 280 °C; carrier gas helium (1 mL/min); split mode (1:200); and volume of injection 1 mL (4% essential oil/CH_2_Cl_2_ *v*/*v*). The heating ramp was as follows: 60 °C for 1 min, 60–280 °C at 10 °C/min, then 280 °C for 1 min. The relative percentages of compounds in each essential oil were determined from the peak areas in the GC–FID profiles. The mass spectrometer operating conditions were as follows: ionization at 70 eV and an ion source temperature of 180 °C. Mass spectral data were acquired in the scan mode in the m/z range of 40–400. Oil solutions were injected with the split mode (1:96) [49].

The identity of components was determined, comparing their retention indices relative to the C_9_–C_22_ *n*-alkanes on the SPB-5 column, computer matching of spectral MS data with those from National Institute of Standard and Technology (NIST) Mass Spectral Library (MS) 107 and 21 [50] and the comparison of the fragmentation patterns with those reported in the literature [51].

### 4.2. Preparation of Essential Oil-Loaded Nanocapsules (EO-NCs)

The EO-loaded nanocapsules were prepared according to the preparation described in Granata et al. [28], but with slight changes, above all concerning scaling up by about three times. A solution of sorbitan monostearate (112 mg), PCL (320 mg), and EO (1.0 g) in acetone (80 mL) at 30 °C was poured into an aqueous solution of polysorbate 80 (275 mg in 160 mL of pure water) while stirring. The suspension was stirred for another 10 min at 25 °C. Then, the organic solvent was removed carefully under vacuum conditions (bath at 30 °C, pressure gradually reduced from 550 to 100 mbar in 1 h, and then reduced at 90 mBar and kept constant for 10 min). Finally, in order to complete the solvent evaporation, the mixture was fluxed by N_2_ (1 h, atmospheric pressure), obtaining the EO-NC suspension (160 mL). The empty NC suspension was achieved under the same conditions but without essential oil.

### 4.3. Physicochemical Characterization of EO-NCs

#### 4.3.1. Encapsulation Efficiency (EE) and Loading Capacity (LC) of EO-NCs

The total amount of essential oil in the EO-NC suspensions was determined by UV−vis spectroscopy (8453 UV-Visible Spectrophotometer, Agilent Technologies, Milan, Italy) over wavelengths ranging from 250 to 450 nm (λ_max_ 274). A 20 μL EO-NC suspension was diluted with 2 mL of acetonitrile, and the absorbance at 274 nm was registered and corrected from the small absorbance due to the other components. The OV or TC concentration was determined from the value of the correct absorbance compared to the linear calibration curve of respective EO, obtained by plotting the absorbance at 274 nm of twelve solutions containing different concentrations of OV (from 11 to 118 µg/mL, R^2^ = 0.9999) or eleven solutions containing TC (from 10 to 102 µg/mL, R^2^ = 0.9999). The ultrafiltration centrifugation technique (Nanosep 30K Omega, Pall Life Science, Milan, Italy; 90 min at 3500× *g*) was used to estimate the free essential oil. The suspension (500 µL) of the EO-NC was ultrafiltrated, and then an aliquot (60 µL) of the filtrate was diluted with 2 mL of acetonitrile. As described above, the free essential oil amount was determined by the absorbance at 274 nm of the resulting solution. From the total and free amounts of essential oil, the encapsulation efficiency was calculated:

EE (%) = ([EO]_tot_ − [EO]_free_)/[EO]_tot_ × 100 (1)

where [EO]_tot_ and [EO]_free_ are the total and free amounts of essential oil in the EO-NC suspensions, respectively.

The loading capacity was calculated by the following equation:

LC (%) = (mass of loaded EO)/(mass of loaded nanocapsules) × 100(2)

#### 4.3.2. Particle Size, Polydispersity and Zeta Potential Measurements

The mean diameter (z-average), the polydispersity index (PDI) and the I-weighted distribution of the EO-NCs were obtained at 25 °C by dynamic light scattering (DLS). The zeta potential (*ζ*) values were determined by electrophoretic light scattering (ELS). DLS and ELS experiments were performed on a Zetasizer Nano ZS-90 (Malvern Instruments, Cambridge, UK), and data were analyzed using Zetasizer Version 7.02 software. For these purposes, the EO-NC suspensions were previously diluted (1:200, *v*/*v*) with pure water or with a pre-filtered (0.45 µm) 10 mM NaCl aqueous solution to carry out DLS or ELS, respectively.

Each experiment was replicated at least twice, and measurements performed at least three times. All data are expressed as mean ± standard deviation (SD).

### 4.4. Microorganisms and Growth Conditions

The fungal strains used in this study, their environment of isolation and sources are detailed described in Table 3 (*Aspergillus fumigatus*, *Aspergillus flavus*, *Penicillium rubens*, *Penicillium citrinum*, *Cladosporium aggregatocicatricatum*, *Cladosporium herbarum*, *Fusarium oxysporum*, *Geotrichum candidum*, *Mucor circinelloides*, *Exophiala xenobiotica*, *Purpureocillium lilacinum*, *Pleurotus eryngii* CCBAS471, *Bjerkandera adusta* CCBAS232 and *Phanerochaete chrysosporium* CCBAS570). The fungal strains were grown at 26 °C on malt extract agar (MEA) for 5 days.

### 4.5. Minimum Inhibitory Concentration (MIC) and Minimum Fungicidal Concentration (MFC)

The MIC and MFC values of the nanoencapsulated essential oils (EO-NCs) and pure EOs were evaluated with a panel of fourteen fungal strains (Table 3). Fungal suspensions were prepared according to de Lira Mota et al. [52] by washing the surface of the MEA slant culture with 5 mL of sterile saline and shaking the suspensions for 5 min. The resulting mixture of sporangiospores and hyphal fragments was withdrawn and transferred to a sterile tube. After heavy particles were allowed to settle for 3–5 min, the upper suspension was collected and vortexed for 15 s. Fungal suspensions were adjusted to a final concentration of 10^6^ conidia mL^−1^ in malt extract broth (MEB). Nine milliliters (9 mL) of MEB containing one hundred microliters of the fungal suspension was distributed into incubation flasks. Encapsulated essential oils of oregano (OV-NC) and thyme (TC-NC) at different concentrations (0.05 to 0.5 mg/mL) and pure oregano (OV-EO) and thyme (TC-EO) at concentrations ranging from 0.05 to 5 mg/mL were added individually to the flasks. The flasks were then incubated at 26 °C for 7 days. After incubation, those fungal strains that did not show any growth were transferred to fresh MEA plates without EO-NCs for an additional 7 days at 26 °C to confirm which concentration had a fungicidal effect. The concentration unfavorable for growth revival during the transfer experiment was taken as the MFC, and this effect was identified as fungicidal. The lowest concentration of EOs or EO-NCs that prevented visible fungal growth and allowed a revival of fungal growth during the transfer experiment was considered the MIC.

## 5. Conclusions

We prepared essential oil-loaded nanocapsules with broad-spectrum antifungal activity. The obtained results suggest the efficacy of nanoencapsulation for enhancing pure essential oil activity. These ecofriendly nanosytems could be a valid alternative to synthetic antifungals and could find effective applications in various industrial sectors concerning health, agri-food and the environment.

## Figures and Tables

**Table 1:** Chemical composition of commercial *Origanum vulgare* and *Thymus capitatus* essential oils (EOs).

# ^a^	RI Lit ^b^	RI Exp ^c^	Class or Compound ^d^	*O. vulgare*^e^ % ^f^	*T. capitatus* % ^f^
			**Monoterpene Hydrocarbons**	**27.99**	**22.26**
1	930	924	α-Thujene	0.07	0.61
2	939	931	α-Pinene	1.37	0.79
3	954	947	Camphene	0.56	0.16
4	979	974	β-Pinene	0.43	N.D.
7	991	986	β-Myrcene	0.66	1.29
9	1002	999	Δ2-Carene	0.06	N.D.
10	1003	1003	α-Phellandrene	N.D.	0.18
11	1004	1009	*p*-Mentha-1(7),8-diene	N.D.	0.07
12	1017	1013	α-Terpinene	0.36	1.03
13	1025	1027	*p*-Cymene	21.54	9.26
14	1029	1029	Limonene	0.78	0.46
16	1060	1058	γ-Terpinene	2.16	8.22
18	1089	1085	Terpinolene	N.D.	0.19
			**Oxygenated Monoterpenes**	**68.66**	**72.88**
15	1031	1031	1,8-Cineole	0.84	0.13
17	1070	1067	*cis* Sabinene hydrate	N.D.	0.14
19	1097	1098	Linalool	4.26	0.86
20	1146	1144	Camphor	0.36	N.D.
21	1156	1158	Isoborneol	0.27	N.D.
22	1169	1167	Borneol	0.90	0.41
23	1177	1177	Terpinen-4-ol	0.35	0.59
24	1189	1190	α-Terpineol	0.61	N.D.
25	1208	1238	*trans*-Piperitol	N.D.	0.06
26	1245	1243	Carvacrol methyl ether	0.16	0.06
27	1253	1259	Geraniol	N.D.	0.08
28	1290	1303	Thymol	25.02	0.58
29	1299	1319	Carvacrol	35.95	69.91
31	1373	1364	Carvacrol acetate	N.D.	0.06
			**Sesquiterpenes**	**1.94**	**3.19**
32	1419	1425	β-Caryophyllene	1.70	2.56
33	1455	1459	α-Humulene	0.24	0.10
34	1506	1492	β-Bisabolene	N.D.	0.20
35	1507	1525	(*Z*)-α-Bisabolene	N.D.	0.16
36	1583	1569	Caryophyllene oxide	N.D.	0.23
			**Others**	**0.47**	**0.22**
5	979	976	1-Octen-3-ol	0.21	0.16
6	984	982	3-Octanone	0.10	N.D.
8	978	993	3-Octanol	0.05	N.D.
30	1359	1361	Eugenol	0.11	0.06
			**TOTAL**	**99.06**	**98.55**

^a^ The numbering refers to the elution order. ^b^ Literature retention index (RI). ^c^ Retention index (RI) relative to the standard mixture of *n*-alkanes on the SPB-5 column. ^d^ Identified compounds (those < 0.05% have not been reported). ^e^ Data previously reported (see [27]). ^f^ Relative peak area percent, representing the averages of three determinations. N.D. = not detected.

**Table 2:** Physicochemical Characterization of essential oil (EO)-NCs.

EO-NCs	*Z*-Average (nm)	PDI	ζ (mV)	EE%	LC%
TC-NCs	198 ± 3	0.09 ± 0.02	−11 ± 1	84 ± 6	52 ± 3
OV-NCs	200 ± 3	0.05 ± 0.03	−10 ± 2	80 ± 9	51 ± 4

**Table 3:** Characteristics of the assayed fungal strains.

Fungal Strain	Environment of Isolation	Source
*Aspergillus fumigatus*	Indoor air	IMB-SAS
*Aspergillus flavus*	Indoor air	IMB-SAS
*Penicillium rubens*	Indoor air	IMB-SAS
*Penicillium citrinum*	Fruit	IMB-SAS
*Cladosporium aggregatocicatricatum*	Wax seal	IMB-SAS
*Cladosporium herbarum*	Wax seal	IMB-SAS
*Fusarium oxysporum*	Cheese	IMB-SAS
*Geotrichum candidum*	Cheese	IMB-SAS
*Mucor circinelloides*	Cheese	IMB-SAS
*Exophiala xenobiotica*	Soil	IMB-SAS
*Purpureocillium lilacinum*	Stone	IMB-SAS
*Pleurotus eryngii*	Tree	CCBAS
*Bjerkandera adusta*	Tree	CCBAS
*Phanerochaete chrysosporium*	Tree	CCBAS

IMB-SAS = fungal collection of the Institute of Molecular Biology (Slovak Academy of Sciences). CCBAS = Culture collection of Basidiomycetes, Institute of Microbiology, Academy of Sciences of the Czech Republic.

**Table 4:** Minimum inhibitory concentration (MIC) and minimum fungicidal concentration (MFC) values of encapsulated essential oils and pure essential oils.

Fungal Strain	TC-NCs		OV-NCs		TC-EO		OV-EO	
	MIC	MFC	MIC	MFC	MIC	MFC	MIC	MFC
*Aspergillus fumigatus*	0.125	0.25	0.125	0.25	0.25	0.5	0.25	0.5
*Aspergillus flavus*	0.25	0.5	0.25	0.5	0.5	1	0.5	1
*Penicillium rubens*	0.25	0.5	0.25	0.5	0.5	1	0.5	1
*Penicillium citrinum*	0.25	0.5	0.25	0.5	0.5	1	0.5	1
*Cladosporium aggregatocicatricatum*	0.125	0.25	0.125	0.25	0.25	0.5	0.25	0.5
*Cladosporium herbarum*	0.125	0.25	0.125	0.25	0.5	1	0.25	0.5
*Fusarium oxysporum*	0.25	0.5	0.25	0.5	0.5	1	0.5	1
*Geotrichum candidum*	0.25	0.5	0.25	0.5	0.5	1	0.5	1
*Mucor circinelloides*	0.25	0.5	0.25	0.5	0.5	1	0.5	1
*Exophiala xenobiotica*	0.25	0.5	0.25	0.5	0.5	1	0.5	1
*Purpureocillium lilacinum*	0.25	0.5	0.25	0.5	0.5	1	0.5	1
*Pleurotus eryngii*	0.125	0.25	0.125	0.25	0.25	0.5	0.25	0.5
*Bjerkandera adusta*	0.25	0.5	0.125	0.5	0.5	1	0.5	1
*Phanerochaete chrysosporium*	0.25	0.5	0.25	0.5	0.5	1	0.5	1

MIC and MFC are expressed in mg/mL. TC-NCs = nanoencapsulated thyme essential oil; OV-NCs = nanoencapsulated oregano essential oil; TC-EO = thyme essential oil; and OV-EO = oregano essential oil.

## Data Availability

The data presented in this study are available in this article.
